# A simplified lung ultrasound approach to detect increased extravascular lung water in critically ill patients

**DOI:** 10.1186/s13089-017-0068-x

**Published:** 2017-06-13

**Authors:** Antonio Anile, Jole Russo, Giacomo Castiglione, Giovanni Volpicelli

**Affiliations:** 1 Intensive Care Unit, Ospedale Vittorio Emanuele, AOU Policlinico–Vittorio Emanuele, Catania, Italy; 2 Department of Anesthesia and Intensive Care, Ospedale Vittorio Emanuele, ASP Caltanissetta, Gela, Italy; 3 Department of Emergency Medicine, San Luigi Gonzaga University Hospital, Orbassano, Turin, Italy

## Abstract

**Background:**

The quantification of B-lines at lung ultrasonography is a valid tool to estimate the extravascular lung water (EVLW) in patients after major cardiac surgery. However, there is still uncertainty about the correlation between B-lines and EVLW in a general population of critically ill.

**Aim:**

To evaluate a simplified lung ultrasonographic assessment as a tool to estimate the EVLW in critically ill patients admitted to a polyvalent intensive care unit (ICU).

**Methods:**

Nineteen consecutive critically ill patients requiring mechanical ventilation and hemodynamic monitoring were enrolled. Lung ultrasonography and the thermodilution methodology (PiCCO system) were performed by two independent operators. The positive scan at lung ultrasound was defined by visualization of at least 3 B-lines. We then compared the number of chest areas positive for B-lines with the EVLW index obtained by the invasive procedure.

**Results:**

A significant correlation was found between the number of lung quadrants positive for B-lines and EVLW indexed using both actual body weight (*rho* = 0.612 *p* = 0.0053) and predicted body weight (*rho* = 0.493 *p* = 0.032). Presence of more than 3 positive lung quadrants showed a good performance in identifying an EVLW index value >10 ml/kg of actual body weight(area under the ROC 0.894; 95% CI 0.668–0.987 *p* < 0.0001). Presence of of more than 4 positive lung quadrants indentified an EVLW index value >10 ml/kg of predicted body weight (area under the ROC 0.8; 95% CI 0.556–0.945 *p* = 0.0048).

**Conclusion:**

A simplified lung ultrasound approach can by used as a reliable noninvasive bedside tool to predict EVLW in emergency and critically ill patients.

## Background

The early evaluation of haemodynamic status and need for fluid treatment are essential in the management of patients presenting with severe cardio-pulmonary failure, both those who are spontaneously breathing and those who are mechanically ventilated [1].

Accurate assessment and monitoring of haemodynamic status often require the placement of a pulmonary artery catheter or other invasive vascular lines. Trans-pulmonary thermodilution is often used for measuring volumetric haemodynamic parameters, of which the most commonly used are the global end diastolic volume and extra vascular lung water (EVLW) [2, 3]. This procedure is based on a central venous access and femoral arterial line, without the need for pulmonary artery catheterization.

The change in EVLW is a useful parameter for monitoring the evolution of cardiogenic pulmonary oedema, acute lung injury and acute respiratory distress syndrome (ARDS). In previous studies, the EVLW index showed a statistically significant correlation with PaO_2_/FiO_2_ ratio in ARDS patients [4].

In recent years, ultrasonography has shown to be useful as a “new eye” to look inside the lung [5, 6]. A change in the balance between air and fluids in the lung parenchyma determines a significant and corresponding change in the acoustic patterns that can be easily detected and measured.

After studying the artefacts that the air/fluid imbalance creates, it is possible to diagnose some pulmonary conditions, ranging from lung consolidations to an increased interstitial water content [7]. The B-lines are vertical echoic comet-tail artefacts detected by lung ultrasonography, and they are typically correlated with the loss of pulmonary aeration and an increase in lung water [8].

Lichtenstein was the first who published about B-lines as a sign of interstitial lung water in 1997 [9].

In 2004, Jambrik et al. published the first paper showing the use of lung ultrasonography to assess interstitial lung water in the lung [10].

In 2005, Agricola et al. found a good correlation between the number of B-lines at lung ultrasonography and EVLW in a selected group of 20 post-operative cardiac surgery patients [11]. Their data demonstrated, for the very first time, a correlation between lung ultrasonography and invasive measurements of EVLW. However, the limitation of their study was that the data were obtained from only a specific subgroup of post-surgical patients that represents a minority of the population usually submitted to invasive haemodynamic monitoring in the intensive care unit (ICU). Moreover, the ultrasound technique proposed in their study is quite time consuming, which has also been suggested by recent evidence-based recommendations [7].

In the years following these studies, more studies have showed the ability of lung ultrasonography to reliably assess lung water or, in general, the aeration of the lungs [12, 13].

In 2015, Zhao et al. showed a very good correlation between lung ultrasound score (LUS) and EVLW, as measured using trans-pulmonary thermodilution, in a population of 21 ARDS patients [14].

The aim of our study is to evaluate the correlation between B-lines and EVLW in a general population of mechanically ventilated ICU patients. Moreover, a further aim is to assess the possibility of using a very simplified ultrasound approach based on a technique that is easily reproducible at the bedside.

## Methods

### Patients

The study was performed between June and August in 2010 in an 8 bed adult general ICU of the university-affiliated Vittorio Emanuele Hospital in Catania, Italy. All consecutive adult ICU patients requiring mechanical ventilation and haemodynamic monitoring with trans-pulmonary thermodilution in the opinion of the attending physician were enrolled. Our critically ill patients are admitted to the ICU from the emergency department, from post-surgery and post-procedure settings, and from the trauma centre. These patients are referred from all of the ordinary wards in the hospital when their haemodynamic status worsens to a level with the critical need for invasive ventilation. A lung ultrasound study was performed in all patients immediately after they obtained a trans-pulmonary thermodilution haemodynamic profile (PiCCO System). The two methodologies were performed separately by two independent operators. Particularly, the operator performing the lung ultrasound study was totally unaware of the results of the haemodynamic invasive analysis. Lung ultrasonography was performed by an anaesthesiology resident trained in lung ultrasonography under the direct supervision of a senior physician who had more than 5 years of experience in critical care ultrasonography.

Both lung ultrasonography and haemodynamic monitoring are part of routine management protocols in our ICU, and their application was totally independent from the study protocol. All data were treated anonymously, as represented by the standardized protocol applied in our institution for any patient admitted for treatment. The informed consent of patients was waived because the patients were all unconscious and the study did not involve additional procedures to the standardized clinical protocols, apart from the anonymous treatment of data.

### Lung ultrasound

The sonographic examinations were performed with the patients in the supine position. Four quadrants were examined in each hemithorax: anterior–superior, anterior-basal (between the parasternal line and the anterior axillary line), lateral-superior and lateral-basal (between the anterior axillary line and the posterior axillary line). A longitudinally oriented probe was used. In total, 8 quadrants were studied in each patient. As previously defined, the B-lines are an “echoic, coherent bundle with a narrow basis, spreading from the pleural line to the edge of the screen without fading”. When 3 or more B-lines were visualized in a single chest area, the quadrant was defined as “positive,” as defined in recently published guidelines.

The ultrasound examinations were performed using a single ultrasound system (Acuson Cypress, Siemens Medical Solutions USA Inc., Mountain View, CA, USA) equipped with a 5–7 MHz phased-array probe (Acuson 3V2c).

### PiCCO system

The trans-pulmonary thermodilution technique (Pulsion PiCCO Plus, Pulsion Medical Systems, München, Germany) was used for haemodynamic monitoring.

In all patients, a 5 Fr arterial catheter was placed in the femoral artery (PV2015LZQ-A Pulsion Medical Systems, München, Germany), and an 8.5 Fr multi-lumen central venous catheter was placed in the internal jugular vein or subclavian vein (Arrow International, Reading, CA, USA). The position of the central venous catheter was verified using a standard chest radiogram.

For the calibration of the system, 3 injections of 15 ml cold saline were performed. Cardiac output was measured, whereas EVLW was computed by the machine. For the calculation of the EVLW index, the actual and predicted body weights were used. For the predicted body weight (PBW) calculation, the following formulas were used: PBW (kg) = 0.91 (height cm—152.4) +50 (for males) and PBW (kg) = 0.91 (height cm—152.4) +45.5 (for females) [14].

### Statistical analysis

Data are expressed as the median and the range.

The Spearman (rho) rank correlation test was used to analyse the correlation between the number of chest quadrants positive for B-lines at lung ultrasound and the EVLW index values.

Sensitivity and specificity of the number of positive quadrants predictive of the EVLW index >10 ml/kg were adjudicated by using the receiver operative characteristic (ROC) curve. A *p* value of <0.05 was considered statistically significant. The statistical analysis was performed using the software MedCalc version 11.6.1.0 (MedCalc Software, Mariakerke, Belgium).

## Results

Nineteen consecutive adult patients requiring mechanical ventilation and haemodynamic monitoring with trans-pulmonary thermodilution were enrolled in the study.

The patient characteristics are shown in Table 1, and the diagnoses of their admission in the ICU are listed in Table 2.

**Table 1:** Patient characteristics

	Median	Range
Age (years)	61	23–77
Height (cm)	165	155–180
Actual body weight (kg)	74	50–100
Predicted body weight (kg)	60	47–75
APACHE II	15	10–41
CVP (mmHg)	13.5	7–22
PEEP (cmH_2_O)	10	0–14
Tidal volume (ml/kg)	8	5.7–10
PaO_2_/FiO_2_	164	100–335

**Table 2:** Admission clinical diagnosis

Acute myocardial infarction	1
Septic shock	4
Acute respiratory failure	6
Acute pancreatitis	1
Cardiogenic shock	1
Inhalation pneumonitis	1
Acute on chronic hearth failure	1
Acute pulmonary edema following hepatectomy	1
Hemorrhagic shock	1
Pulmonary edema following tricuspid valve plasty	1
Bowel perforation	1

**Fig. 1:**
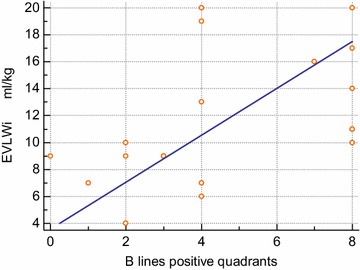
Correlation between the number of chest quadrants positive for B-lines at lung ultrasound and the values of EVLW/kg ABW. Spearman (rho) rank correlation test: sample size—19; Spearman’s coefficient of rank correlation (rho)—0.162; significance level—*p* = 0.0053; 95% confidence interval for rho—0.219 to 0.834

**Fig. 2:**
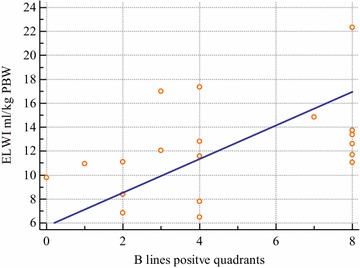
Correlation between the number of chest quadrants positive for B-lines at lung ultrasound and the values of EVLW/kg PBW. Spearman (rho) rank correlation test: sample size—19; Spearman’s coefficient of rank correlation (rho)—0.493; significance level—*p* = 0.032; 95% confidence interval for rho—0.499 to 0.774

In all patients, lung ultrasound examination was feasible. The median number of chest quadrants positive for B-lines was 4 (range 0–8). The median EVLW index was 10 ml/kg actual body weight (ABW) (range 4–20) and 11 ml/kg PBW (range 6–22). A significant correlation was found between the number of positive quadrants at lung ultrasound examination and EVLW index (*rho* = 0.612 and *p* = 0.0053 for ABW; *rho* = 0.493 and *p* = 0.032 for PBW) (Figs. 1, 2).

**Fig. 3:**
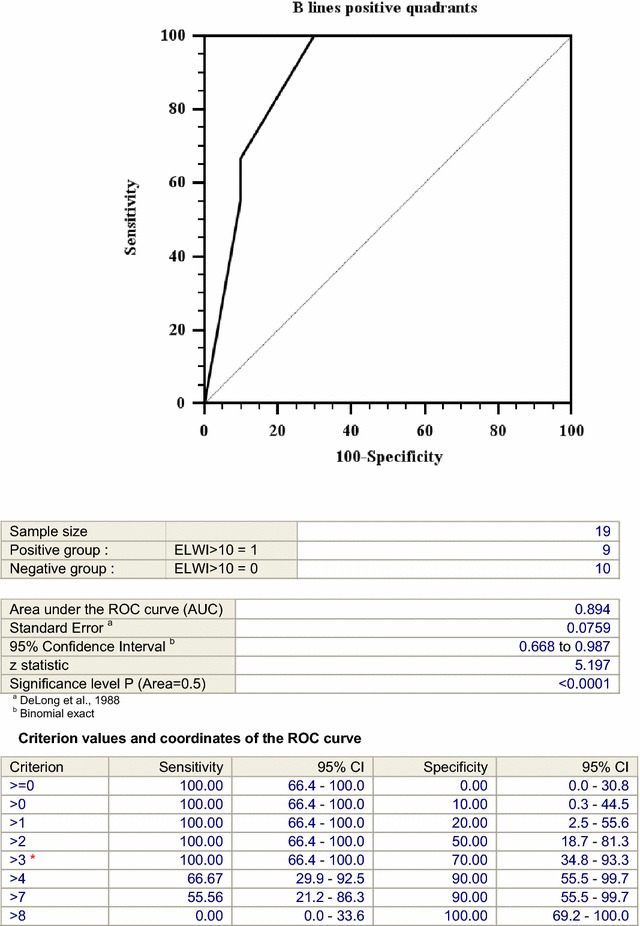
Receiver operative characteristic (ROC) *curve* for EVLW/kg ABW

The detection of more than 3 positive chest quadrants upon lung ultrasound examination showed the best specificity (70%) and sensitivity (100%) for predicting an EVLW index >10 ml/kg ABW (area under ROC curve: 0.894; 95% CI 0.668–0.987, *p* < 0.0001, see Fig. 3).

**Fig. 4:**
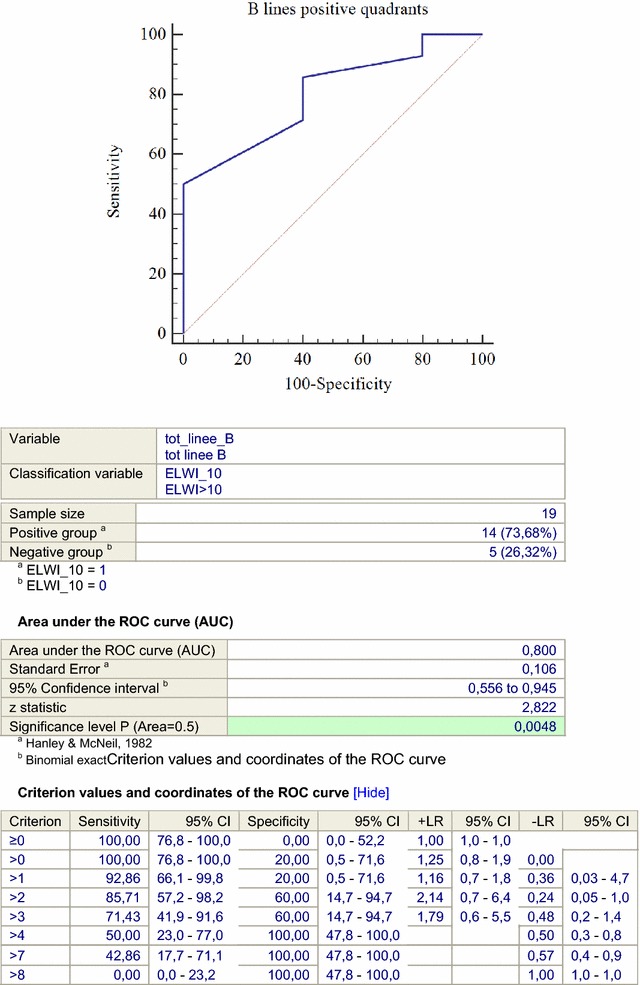
Receiver operative characteristic (ROC) *curve* for EVLW/kg PBW

Detection of more than 4 positive chest quadrants at lung ultrasound examination showed the best specificity (100%) and sensitivity (50%) for predicting an EVLW index >10 ml/kg PBW (area under ROC curve: 0.8; 95% CI 0.556–0.945, *p* = 0.0048, see Fig. 4).

## Discussion

Our study confirms the correlation between B-lines and EVLW in critically ill patients with various haemodynamic conditions and diseases. Moreover, we found that the presence of more than 3 B-line positive chest quadrants correlates well with EVLW superior to the threshold of normality when EVLW is indexed for the actual body weight. When using the predicted body weight, as is what is reported in some papers in the literature, the number of B-line positive quadrants that best predicts EVLW >10 ml/kg was 4.

In recent years, ultrasonography has been increasingly used in daily practices in the ICU and has showed its usefulness in monitoring the pulmonary aeration and haemodynamic condition of critically ill patients [14, 15]. Haemodynamic evaluation using a bedside ultrasound offers a large number of advantages to invasive monitoring, including the absence of risks, not needing costly disposable devices, and rapid and easy repeatable assessments.

Some studies demonstrated the reliability of ultrasound evaluation in detecting lung oedema.

A seminal study by Agricola et al. showed a good correlation between lung ultrasound B-lines and EVLW measured by trans-pulmonary thermodilution. This method is widely used in haemodynamic monitoring and is minimally invasive in comparison to pulmonary artery catheterization because of its capability to measure not only “pressures” but also “volumes.” Extravascular lung water is one of the many important parameters that become available when this technique is applied at the bedside, and it is universally considered as one of the most important parameters to predict the real haemodynamic status and response to treatment of the critically ill. The potential of lung ultrasound examination to predict EVLW allows for the hypothesis that it can be used as an alternative to invasive procedures when the PiCCO methodology is not yet available at the bedside, as it usually occurs in the emergency setting before admission to the ICU. The study of Agricola et al. already demonstrated the correlation between the invasive EVLW and B-lines, but it evaluated only a selected population of post-operative cardiac surgery patients and excluded patients with lung diseases. In our opinion, this selection prevents the generalization of the results of the study to other patient populations. In a similar study, our group assessed the correlation between a positive lung ultrasound examination for B-lines and high EVLW or wedge pressure in a wide and multifaceted population of critically ill patients using a multi-centre enrolment scheme [16]. While Agricola et al. used a time consuming chest count of B-lines to find the linear correlation with EVLW, Volpicelli et al. applied only a dichotomous evaluation based on the positivity of lung ultrasound evaluation for B-lines, without investigating a numeric correlation between the artefacts and EVLW values.

Our study was designed to evaluate a general ICU population requiring mechanical ventilation and haemodynamic monitoring, without exclusion criteria. For this specific reason, but compatible with our sample size, our results may be better generalized to the real practice of a polyvalent ICU and in general to emergency and critical care medicine. Moreover, the ultrasound technique that we applied in our study, the simple counting of positive chest quadrants, is less labourious than Agricola’s technique but still allows for the analysis of a linear numeric correlation between the artefacts and EVLW values.

We chose 10 ml/kg as a cut-off value for an elevated EVLW index, which is in agreement with previous literature [17]. Only one of our patients with an EVLW index >10 ml/kg showed a PaO_2_/FiO_2_ >200. This observation is a further demonstration that EVLW has a very close association with clinically relevant gas exchange impairment.

One practical application of our limited fast lung ultrasound examination at the bedside may be in the extreme emergency setting, when the critically ill patients are still in the very early initial phase of diagnostic evaluation. In this situation, lung ultrasound examination may be very helpful in confidently ruling out the condition of elevated EVLW, possibly allowing the treating physician to be more confident in applying an intensive fluid treatment, when indicated, and in monitoring the effect on pulmonary function [18]. The new appearance of B-lines during this early phase may be considered a sign of rapidly increasing EVLW, which is a condition that demands for a stop in fluid administration [19]. On the other hand, a positive lung ultrasound examination with more than 3 or 4 positive quadrants at the very early stage should lead to a more careful consideration of the haemodynamic state before any definitive conclusion is made on the fluid treatment.

A great limitation of our study is the relatively low number of patients enrolled. The significance of our results should be read under this limited perspective. However, while we cannot exclude that increasing the number of enrolled patients may have a significant influence on the best numeric correlation between the lung ultrasound cut-off and elevated EVLW, our data represent a further confirmation of the tight relationship that exists between B-lines and EVLW detected by the PiCCO technique.

## Conclusion

In conclusion, we showed a correlation between the number of lung quadrants positive for the presence of B-lines at lung ultrasound examination and extravascular lung water. We also showed that the detection of more than 3 B-line positive chest quadrants, when using ABW for indexing EVLW, or more than 4 B-line positive chest quadrants, when using PBW, allows for the prediction of abnormal EVLW value, with strong sensitivity and specificity. Results from our study support the use of lung ultrasonography as a screening tool in the emergency and critical care settings with an acceptable performance at the bedside in identifying patients without a significant condition of increased EVLW, with encouraging rapidity and reliability.
